# Self-produced audio-visual animation introduction alleviates preoperative anxiety in pediatric strabismus surgery: a randomized controlled study

**DOI:** 10.1186/s12886-021-01922-6

**Published:** 2021-04-07

**Authors:** Yuexi Jin, Aifen Jiang, Wanna Jiang, Wenxin Wu, Lisha Ye, Xiaojiang Kong, Le Liu, Zhousheng Jin

**Affiliations:** 1 Department of Anesthesiology, Eye Hospital and School of Ophthalmology and Optometry, Wenzhou Medical University, Xueyuan Road #270, Wenzhou, Zhejiang China; 2 Wenzhou Medical University, Wenzhou Chashan Senior education park, Ouhai District, Wenzhou, Zhejiang China; 3 Department of Anesthesiology, The First Affiliated Hospital of Wenzhou Medical University, South Baixiang Town, Wenzhou, Zhejiang China

**Keywords:** Audio-visual animation, Anxiety, Pediatric strabismus surgery, Parents, Agitation

## Abstract

**Background:**

Hospital anxiety caused by strabismus surgery has an unpleasant and disturbing feeling for both children and their parents. This study aimed to determine the effect of viewing a self-produced audio-visual animation introduction on preoperative anxiety and emergence agitation of pediatric patients undergoing strabismus surgery.

**Methods:**

In this prospective randomized study, 1 hundred children scheduled for strabismus surgery with aged 3 ~ 6 years. The children were randomly divided into 2 groups (*n* = 50 for each), Group A: using a self-produced audio-visual animation introduction; Group C: controlled group without audio-visual animation introduction. Children’s preoperative anxiety was determined by the modified Yale Preoperative Anxiety Scale (mYPAS) at different time points: the night before surgery(T1), at pre-anesthetic holding room(T2), and just before anesthesia induction(T3). The Spielberger State-Trait Anxiety Inventory (STAI) was used to record the anxiety of parents at T1,T2 and T3. The incidence and the degree of emergence agitation were recorded.

**Results:**

The mYPAS scores at T2 and T3 were higher than T1(*p* < 0.05) in both groups. The average score of mYPAS in Group A was significantly lower than in Group C at T2 and T3(*p* < 0.05). The STAI scores in Group A at T2 and T3 were significantly lower than in Group C(*p* < 0.05). The incidence of agitation in Group A was lower than that in Group C(*p* < 0.05).

**Conclusions:**

Based on the findings, viewing a self-produced audio-visual animation can effectively alleviate the preoperative anxiety for both children and their parents in pediatric strabismus surgery, and it was effective for reducing emergence agitation as well.

**Trial registration:**

The trial was prospectively registered before patient enrollment at Chinese Clinical Trial Registry (Clinical Trial Number: ChiCTR1900025116, Date: 08/12/2019).

**Supplementary Information:**

The online version contains supplementary material available at 10.1186/s12886-021-01922-6.

## Background

Surgical procedure in children examinations or treatments for pathological condition could induce stress for both preschool children and their parents. The stress could be manifested as fear, anger and, frequently, anxiety [1]. The preoperative anxiety of preschool children may be caused by various reasons, such as the separation from parents, the adverse expectation of surgical procedures, and frequently, non-familiarity with the surgical environments and staffs [2]. The preoperative anxiety of preschool children has been reported to be reflected as agitation, shivering and crying, stopping playing, and breathing deeply.

Severe preoperative anxiety was observed to be related to postoperative agitation, difficulty in anesthetic induction and other negative behaviors [3–5]. The prolonged induction period and delayed postoperative awakening may be due to preoperative anxiety [2]. Kain and Mayes [3] reported that the postoperative pain and an increasing usage of analgesics and sedative drugs was observed in patients with intense preoperative anxiety. Consequently, preschool children, who suffer from intense preoperative anxiety, may develop sleep disorders, and behavioral changes, such as aggression toward authority or nocturnal enuresis [6]. The long-term psychological effects of preoperative anxiety include a negative prejudice for future surgical procedures, separation anxiety and even difficulties in school and socialization [2]. The incidence of preoperative anxiety arising from both psychological and physiological findings was reported between 40 to 60% in preschool children. Therefore, the identification and management of preoperative anxiety in preschool children are full of clinical significance in terms of preventing both physiological and psychological side effects [7].

Clinicians have come up with several ways to address preoperative anxiety in preschool children, such as permission of parents to accompany during anesthetic induction, behavioral preparation programs, music therapy and administering sedatives before surgery [8]. However, studies showed that certain children still experienced preoperative anxiety even with prevention strategies [9].

Preschool children usually enjoy watching animated cartoons. In a lot of cartoon characters, most of Chinese children aged 3 to 6 years especially love a cute tiger named Qiaohu, who guides children to adapt to a new environment and become more braver and stronger. This study was performed to determine the beneficial effects of watching a self-produced Qiaohu animated cartoon repeatedly, which introduced the details of the process of anesthesia and surgery, matters needing attention during anesthesia, operative risk, and the outcome of disease, on preoperative anxiety for both children and their parents in pediatric strabismus surgery.

## Method

The study was approved by the Institutional Review Board of The Eye Hospital of Wenzhou Medical University and adhered to the tenets of the Declaration of Helsinki. Written informed consent was obtained from all the subject’s parents. The study adhered to CONSORT guidelines.

One hundred preschool children, without having previous emergency surgery and anesthetic experience, underwent pediatric strabismus surgery at the Eye Hospital of Wenzhou Medical University between August 2019 and December 2019, ASA physical status I or II, aged 3 to 6 years were recruited. Children were excluded from the study if their parents reported developmental delays, mental retardation, or chronic illnesses of the children. Children’s parents were interviewed to rule out mental illness or cognitive impairment. Subjects were randomly allocated into 2 groups by computer-generated random number, Group A: using a self-produced audio-visual animation introduction; Group C: control group without audio-visual animation introduction.

Subjects were hospitalized 1 day in advance before the surgery. Investigator 1 visited the ophthalmic ward to conduct preoperative interviews and measure baseline anxiety scores. Then, the children in Group A received preoperative education by watching a self-produced audio-visual animation (*n* = 50); The children in Group C received routine anesthesia visits including the introduction of anesthesia process, anesthesia precautions and answering some questions (*n* = 50).

Subject’s preoperative anxiety was determined by the modified Yale Preoperative Anxiety Scale (mYPAS) [10]. The mYPAS score ranged from 23.3 to 100, with a score higher than 30 indicates that the subject does have anxiety. Parent’s preoperative anxiety was measured by the Spielberger State-Trait Anxiety Inventory (STAI) which is mainly used to assess immediate or recent experiences or feelings of fear, tension, anxiety and neuroticism at a specific time or situation [11]. The mYPAS were used to measure the children’s anxiety levels at three time points: the night before surgery(T1), at preanesthetic holding room(T2), and just before anesthesia induction(T3). The STAI were used to measure the parents’ anxiety levels at T1, T2 and T3.

In both groups, Investigator 1 introduced the process of anesthesia and operation the night before surgery. Children and one of their parents completed the assessment of the corresponding anxiety scale (T1). In addition, investigator 2 accompanied the children and their parents watching audio-visual animation three times, 10 min at a time and discuss the process of anesthesia and surgery detail in Group A. All strabismus surgeries were performed in the morning. The children and one of their parents in both groups were assessed for anxiety at pre-anesthetic holding room(T2) by investigator 3. The children were accompanied by their parents and investigator 3 during the transportation from the preanesthetic holding room to the OR. The final mYPAS and STAI measurements were taken at T3 by investigator 4. The mYPAS and STAI scores were measured respectively by same investigator at each time point to exclude interrater bias. Meanwhile, investigator 1, investigator 3 and investigator 4 were blinded to the study group.

Solid food and sugar juice were withheld for 8 h and 3 h before surgery, and no preoperative medication was used. After entering the OR, non-invasive blood pressure, electrocardiogram and SpO2 were monitored and intravenous channels were opened. After induction of anesthesia with propofol 2.5 mg/kg, fentanil 2 g/kg and vecuronium 0.1 mg/kg by investigator 5, endotracheal intubation was completed 2 min later and mechanical ventilation was performed. Paw 10 ~ 15 cmH2O was administered with Drager Primus anesthesia machine. Respiratory frequency was adjusted to maintain PETCO233 ~ 43 mmHg. Anesthesia was maintained by inhaling 3% ~ 4% sevoflurane, oxygen 2.0 L/min, BIS value was maintained at 40 ~ 50, and the concentration of sevoflurane exudated 30 min after the operation was started was recorded. Sevoflurane inhalation was stopped 5 min before the end of surgery. Postoperative recovery of spontaneous breathing, tidal volume of more than 8 ml/kg, SpO2 > 95%, PETCO2 < 45 mmHg after breathing fresh air for 5 min, partial recovery of swallowing reflex, tracheal catheter was removed after sputum aspiration and sent to post-anesthetic care unit (PACU).

Emergence agitation was graded by the PACU nurse, who was blinded to the study group, using the following scale: 0 = somnolence and unwakefulness; 1 = conscious, quiet and cooperative; 2 = crying, need to be consoled; 3 = agitated, crying seriously, unable to be consoled, but do not need to be restrained; 4 = fidgety, combative, and disoriented, need to be pressed and braked; ≥2 is defined as agitation, 2 is mild agitation, 3 is moderate agitation and 4 is severe agitation [12].

### Statistical analysis

All data were analyzed using SPSS 19.0 statistical software (SPSS Inc., Chicago, IL). The Shapiro–Wilk test was used for normal distribution test. Data are presented as mean ± standard deviation. Frequencies were used in categorical variables. Patients’ characteristics and anxiety scores between groups were analyzed using two sample t-test that were normally distributed or the Kruskal-Wallis test for variables that had nonparametric data or were not normally distributed. Anxiety scores within groups were analyzed using repeat-measures ANOVA followed by the Holm-Sidak test for multiple comparisons. ASA level, gender, and the incidence of agitation between two groups were analyzed with Fisher exact test. A *P* value < 0.05 was regarded as statistically significant.

## Results

The patient demographic and clinical data in age, ASA level, gender, weight, and height of the patients showed no significant differences between two groups. (*p >* 0.05, see Table 1). The differences in age, gender, marital status, educational background of the parents between two groups were not statistically significant (*p >* 0.05, see Table 2). The STAI scores of the parents in Group A were lower than that in Group C (*p* < 0.05) at T2 and T3, see Table 3 for details. The mYPAS scores of patients in two groups during perioperative period were showed in Table 4. In Intra-Group Comparison, the mYPAS scores at T2 and T3 were higher than those at T1 in both groups (*p* < 0.05); In Group Comparison, there was no statistical significance between Group A and Group C in mYPAS scores at T1, however, the mYPAS scores of Group A at T2 and T3 were significantly lower than those in Group C (*p* < 0.05). The incidence of agitation in Group A was 8%, while that in Group C was 38%. The incidence of emergence agitation in Group A was lower than that in Group C (*p* < 0.05, as shown in Table 5).

**Table 1:** The Patient Demographic and Clinical Data in Two Groups (*n* = 50)

Group	Age (year)	ASA Level (I/II)	Gender (M/F)	Weight (kg)	Height (cm)
C	4.72 ± 1.03	47/3	27/23	18.53 ± 2.59	105 ± 7
A	4.65 ± 1.81	46/4	26/24	18.75 ± 3.15	106 ± 8

**Table 2:** The Demographic and Clinical Data for Children’s Parents in Two Groups (*n* = 50)

Group	Age (year)	Gender (M/F)	Marital Status (in marriage /divorce)	Educational Background (college/high school)
C	30.78 ± 1.62	35/15	50/0	48/2
A	30.95 ± 1.44	34/16	50/0	49/1

**Table 3:** The STAI Scores of the Parents in Two Groups during Perioperative Period (*n* = 50)

Group	T1	T2	T3
C	40.6 ± 6.28	45.3 ± 9.75	47.4 ± 13.4
A	39.4 ± 7.06	40.2 ± 10.45^▲^	43.3 ± 8.58^▲^

Compared with Group C, ^▲^*p* < 0.05

Significant difference noted between T1 versus T3, T2 versus T3, T2 versus T3 in Group A, *p* < 0.05; Significant difference noted between T1 versus T2, T1 versus T3, T2 versus T3 in Group C, *p* < 0.05

**Table 4:** The mYPAS Scores of Children in Two Groups during Perioperative Period (*n* = 50)

Group	T1	T2	T3
C	35.6 ± 6.28	45.3 ± 9.75*	45.4 ± 13.4*
A	35.4 ± 7.06	38.2 ± 10.45^▲^*	42.3 ± 8.58^▲^*

Compared with Group C, ^▲^*p* < 0.05; Compared with T1, * *p* < 0.05

**Table 5:** Comparison of the degree of emergence agitation between the two groups(*n* = 50)

Group	No agitation	Mild	Moderate	Severe
C	31	13	5	1
A^▲^	46	2	2	0

Compared with Group C, ^▲^*p* < 0.05

## Discussion

This study applied a self-produced audio-visual animation, which were recorded videos taking hospital as background, during perioperative period as a behavioral intervention program to effectively reduce preoperative anxiety in pediatric patients underwent strabismus surgery. In this study, Group A with audio-visual animation application presented significantly lower mYPAS scores compared with Group C who only received routine anesthesia visits. In addition, parents’ STAI scores in Group A were significantly reduced compared with Group C.

Our self-produced video was different from the ordinary cartoon [13]. This cartoon created by us took the ward and operating room of our hospital as the background to illustrate the operation and anesthesia. In the video, Qiaohu, a cartoon character, always accompanied the children and their parents to experience the procedure of operation and anesthesia, during which the children were encouraged and explained for the procedure. The whole cartoon was shown to the children three times, 10 min at a time. The reason why we repeat the video is that in the pre-experiment, if children only watched it once, they would not be impressed enough. It worked best when they watched it three times, and watching animation 10 min at a time was less likely to fatigue.

Although preschoolers (4–6 years old) already have a certain development in emotion and feeling, their psychology and behavior are mainly controlled by external stimulation and their own subjective emotion, thereby they have limited ability of self-control. Researches [14–16] showed that pediatric patients were always in a state of excessive psychological stress in perioperative period, which did not only affect the performing of anesthesia and operation, but also delayed postoperative rehabilitation and damaged the physical and mental health of pediatric patients. It was estimated that more than 5 million children performed elective surgery every year, nearly 75% of them appeared different degrees of preoperative anxiety [17]. The preoperative anxiety and fear of children may lead to short-term euphoria, haziness of spirit-affect, dismal, learning disability and sleep disorders after operation, even a long-term serious psychological problem [18]. Therefore, more attention has been paid to the preschoolers’ psychological stress state in perioperative period and efforts have been made to establish a good relationship between doctors and patients. From the results of intra-group comparison in Table 4, the mYPAS scores at T2 and T3 were higher than the scores at T1 in both groups, which indicated that there was an obvious psychological fluctuation in pediatric patients when they had to leave parents to face the unfamiliar operating room, medical staff and clinical operation alone. In group comparison, the average scores of mYPAS in Group A at T2 and T3 were lower than those in Group C (*p* < 0.05), which demonstrated that it could be of great help for relieving pediatric patients’ preoperative anxiety by watching audio-visual materials three times at the time of preanesthetic visit.

Traditional description or expository communication via assisted static images may not make children fully understand the anesthesia and surgery, or even aggravating children’s psychological burden since they are too abstract for children to understand. Currently, anesthesia induction in children with parents’ presence was a popular behavioral intervention based on the research [19], it has been verified that this way could not only relieve the anxiety of children, but also enhanced the compliance of children. However, parents with exceeding preoperative anxious may aggravate children’s anxiety when undergoing anesthesia induction [20]. It was reported that sedative could relive preoperative anxiety, separation anxiety and bad mood during anesthesia induction period in children [7], but pediatric patients may resist venipuncture and refuse medication, which even stimulating abnormal behaviors like dysphoria, delirium, and postoperative changes in behavior, eventually prolonging the healing time after operation [8]. In addition, sedative may cause other side effects, such as respiratory depression, airway obstruction without nursing, increase of medical fees, additional nursing staff and equipment, and delayed recovery [9, 21]. To reduce the rate of above disadvantages, a variety of methods have been attempted. For example, some people changed common administration routes like oral and intravenous to intranasal [22]. Other attempted methods include parents’ presence during induction of anesthesia [23], music, maternal voice [24], toys [25] and education with pamphlets or videos [26]. However, it was revealed that these methods have limited benefits and effectiveness. Kain et al. [26] have developed a structured family-centered behavioral intervention program that could relieve anxiety in children and their parents as well as led to better results than sedative premedication in the incidence of postoperative delirium, consumption of analgesics, and duration of postoperative hospital stay. A new therapy for reliving children’s negative emotion and bad behavior emerged. Cognitive Behavioral Therapy (CBT) is a short-term psychotherapy carried out worldwide for correcting wrong cognition and eliminating the negative feelings and bad behavior by changing the patients’ medical-related beliefs and cognitive model [1]. Most of doctors showed it in Power Point form or videos, few doctors use recorded video in the background of the hospital perioperative period. The audio-visual animation video in this study was a recorded video during perioperative period, in which we used a cartoon figure that was popular among preschoolers to explain the whole process and gave children encouragement. This video tried to change and improve children’s behavior by intuitively connecting the environment in video and similar events. In this study, we found that the preoperative anxiety and incidence of postoperative agitation in children was reduced after preoperative video psychological intervention, which consistent with previous research results.

Salhotra [6] reported that the cognition status and emotion state of parents influenced children’s emotional response to varying degrees. Parents’ overanxious reaction can be perceived by pediatric patients, which may aggravate the children’s anxiety [27]. Therefore, to solve the mental behavior problems of children, their parents’ cognitive and psychological state should be fully taken into consideration. In this study, Table 3 showed that the STAI scores of Group C at T2 and T3 were higher than Group A, which represents that it was effective to take psychological intervention measures on parents. The anxiety of parents during perioperative period is a common symptom that caused by two major factors:①the lack of understanding on medical-related information: they are not familiar with the process of anesthesia and surgery, and the environment of operating theater as well. Also, they are afraid that children can’t face the anesthesia and surgery independently. ②the lack of trust in medical staff: Due to the fast-paced work, parents are afraid that the medical staff may ignore to pacify the mental feelings of children [28]. The effects of CBT on parents through our self-produced audio-visual animation video includes three aspects: 1. Be familiar with environment, from the preanesthetic holding room to the operating room that pediatric patients could see, which was conveyed to parents from children’s perspective. At the meantime, with the cooperation of parents, the doctor could find what the children were interested in or repulsive to use; 2. To feel the scene of interaction between medical staff and pediatric patients, thus eliminating the unnecessary anxiety; 3. To know the whole process of anesthesia, thereby relieving parents’ anxiety to some extent and it also helps to ease children’s mood.

In accordance with the characteristics of preschoolers, such as hyperactive, curious, like imitating and to be praised, this video used Qiao Hu, children’s favorite cartoon character, to guide preschoolers by encouraging and praising them, aiming at reaching a certain degree of regulation and control of preschoolers’ behavior. The audio-visual animation video that took the hospital as shooting background, the cognitive communication with children and their parents at preanesthetic visit, the positive atmosphere created by parents during perioperative period and the imitating the behaviors of Qiao Hu in video, all these strategies made the children better adapt the anesthesia induction, helping them establish a positive attitude to being in hospital and helping doctors complete all invasive clinic manipulation smoothly and strive for good clinic effects. In addition, this kind of psychological intervention can be regarded as one way to improve doctor-patient relationship and patient satisfaction, the psychological communication makes patients and their families know more about medical model, both doctors and patients can understand each other emotionally, this is of great help for hospital to have a good social effect and prevent medical disputes.

## Conclusions

Based on the findings, viewing a self-produced audio-visual animation can effectively alleviate the preoperative anxiety for both children and their parents in pediatric strabismus surgery, and it was effective for reducing emergence agitation as well.

## Supplementary Information

**Additional file 1.**

## Data Availability

The datasets used and/or analyzed during the current study are available from the corresponding author on reasonable request.

## Abbreviations

mYPAS: Modified Yale Preoperative Anxiety Scale
STAI: State-Trait Anxiety Inventory
OR: Operation room
PACU: Post-anesthetic care unit
CBT: Cognitive Behavioral Therapy
